# Supplementary material for: Seminoma and Embryonal Carcinoma Footprints Identified by Analysis of Integrated Genome-Wide Epigenetic and Expression Profiles of Germ Cell Cancer Cell Lines
Source: PLoS One. 2014 Jun 2;9(6):e98330. doi: 10.1371/journal.pone.0098330 (PMC4041891; doi:10.1371/journal.pone.0098330)
Supplement: File S1 — ZIP file containing DMRforPairs output for significant regions. Please start from the html files. (ZIP) [file pone.0098330.s008.zip › figures/27947.pdf]

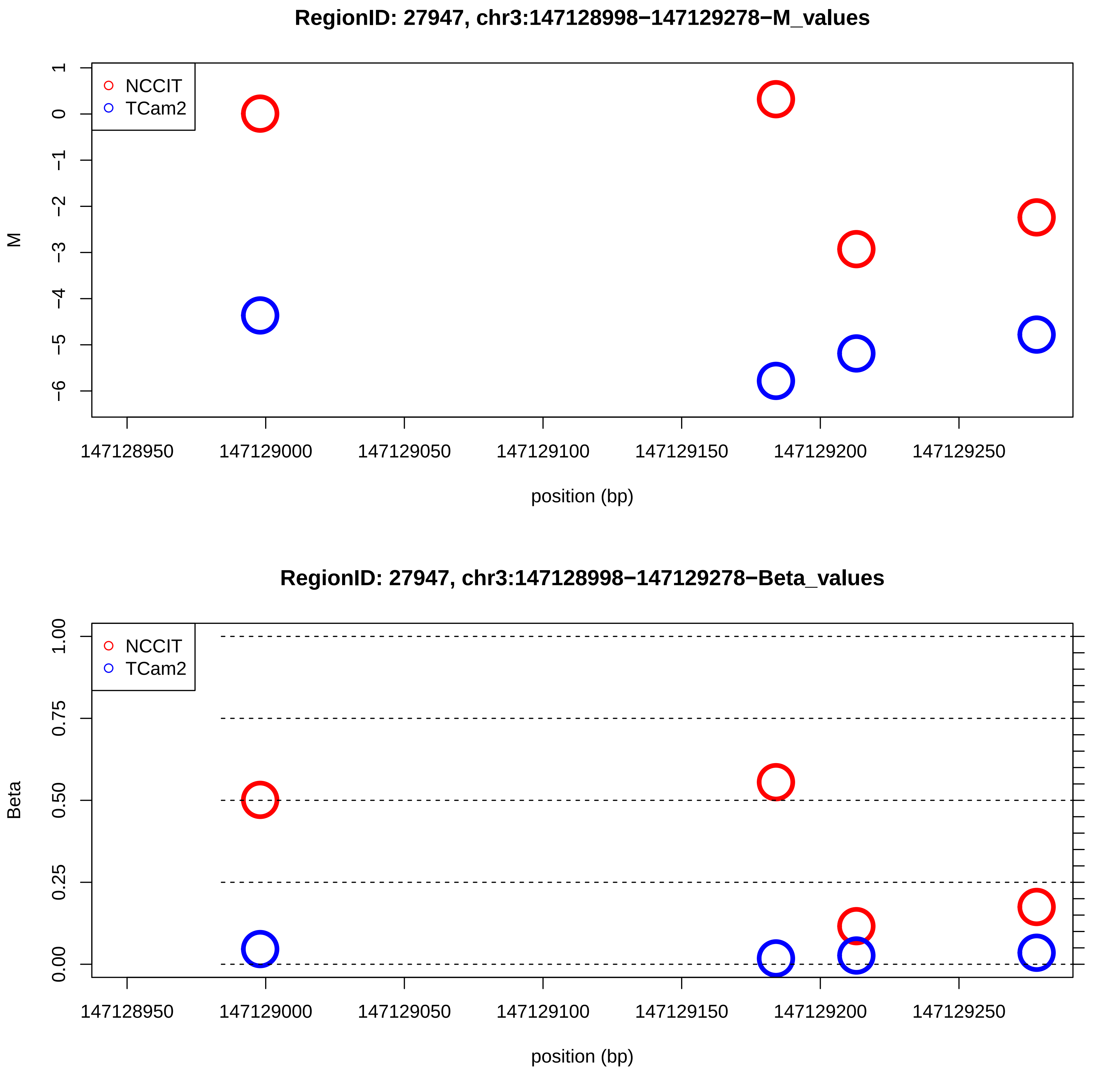

RegionID: 27947, chr3:147128998−147129278−M_values
M
NCCIT
TCam2
1
0
−1
−2
−3
−4
−5
−6
147128950
147129000
147129050
147129100
147129150
147129200
147129250
position (bp)
RegionID: 27947, chr3:147128998−147129278−Beta_values
Beta
NCCIT
TCam2
1.00
0.75
0.50
0.25
0.00
147128950
147129000
147129050
147129100
147129150
147129200
147129250
position (bp)
